# Stability, the Last Frontier: Forage Yield Dynamics of Peas under Two Cultivation Systems

**DOI:** 10.3390/plants11070892

**Published:** 2022-03-27

**Authors:** Vasileios Greveniotis, Elisavet Bouloumpasi, Stylianos Zotis, Athanasios Korkovelos, Constantinos G. Ipsilandis

**Affiliations:** 1 Hellenic Agricultural Organization Demeter, Institute of Industrial and Forage Crops, 41335 Larissa, Greece; 2 Department of Agricultural Technology, Technological Educational Institute of Western Macedonia, 53100 Florina, Greece; 3 Department of Agricultural Biotechnology and Oenology, International Hellenic University, 66100 Drama, Greece; elisboul@abo.ihu.gr; 4 Directorate of Water Management of Thessaly, Decentralized Administration of Thessaly—Central Greece, 41335 Larissa, Greece; athanasios.korkovelos@apdthest.gov.gr; 5 Regional Administration of Central Macedonia, Department of Agriculture, 54622 Thessaloniki, Greece; ipsigene@gmail.com

**Keywords:** low-input, AMMI, GGE biplot, trait stability index, pea (*Pisum sativum* L.)

## Abstract

The stability of performance may be proved to be the last frontier for adopting certain genotypes in various cultivation systems and environments. The main objective of the present study was to analyze the forage yield stability performance of seven pea (*Pisum sativum* L.) genotypes based on various stability indices. The genotype behavior was studied based on the yield of peas under both conventional and low-input cultivation systems. Five cultivars of peas (broadly distributed) and two lines were used in a strip-plot design. Significant positive correlations were detected between forage yield and some other traits. This way, forage yield stability may be indirectly improved by improving certain traits showing qualitative inheritance. Comparisons revealed that genotypes exhibited stable performance, even in low-input farming systems. AMMI analysis, GGE biplot, and analysis of variance (ANOVA) combination showed statistically significant differences between genotypes and environments and the farming system. Our analysis depicted specific cultivars of peas for different areas and farming systems to attain highly stable performance. Vermio was confirmed to be a stable genotype for forage yield performance in low-input farming in Trikala and Kalambaka areas, while Pisso was indicated as the best in Florina and Giannitsa areas in low-input farming. The two pea lines exhibited stable performance in Giannitsa and Florina areas, especially in low-input conditions. The stable behavior of some genotypes in these conditions may be useful for farmers that raise livestock in mountainous areas. The genetic parameters show that the selection for fresh forage yield and dry matter yield in breeding programs is expected to be effective.

## 1. Introduction

The pea (*Pisum sativum* L.) is a very useful crop for supporting livestock [1,2]. Pea cultivations can be found in a wide range of agro-climatic zones; the potential of this crop and its high nutritional value are referred to in many related publications [3,4]. Field pea is often used as a main protein source, as it has a rich and unique protein profile, different from other natural protein sources [5]. On the basis of the aforementioned points, our study focused on estimating the genetic potential needed to support the growth and yield performance of pea cultivations in various environmental conditions and, subsequently, to propose the best cultivars.

The stability of performance could be crucial for adopting certain genotypes in various cultivation systems and environments [6]. Stability may be dependent on cultivar tolerance to various biotic and abiotic factors in certain environments [7]. It is the ability to perform satisfactorily under almost any difficulty and cope with it during the whole growing season. Fasoulas [7], and later Fasoula [8], who used the squared form of reversed Coefficient of Variation as a stability criterion (stability index), tried to describe the plant’s behavior under different circumstances.

Total yield performance is a multi-factor function: genotypes (G), environment (E), and the genotype by environment (GE) [9]. Plant breeders take into account the various implications of GEI in their breeding programs. GEI reduces the correlation between phenotypic and genotypic variability, decreasing heritability [10]. Without GEI analysis, the selection is insufficient for secure gains across selection cycles [11], making stability a high priority in every breeding program. Stability is necessary for the development of new, successful genotypes [12]. Breeders select for positive genotypic stability or try to minimize environmental variability that results in GEI [13].

In peas, many researchers assessed stability using varying approaches and methods, such as G × E classic statistics, cluster, and regression analysis, especially for yield [14,15,16,17], some of them in multi-location environments. Genotype by year interaction was found to be important for morpho-productive traits and biogas production in soybean [18] and in quantitative traits of field pea [19]. All these researchers tried to define the best genotypes suitable for various environments. Genotypes displaying high means of yield components, along with a low degree of fluctuations in different locations or seasons, are considered more adaptive or stable [7,20]. In our novel approach, high adaptability and stability of performance are realized when the stability criterion used shows high values; thus, all methods proper for this analysis must be taken into account. Acikgoz et al. [14] showed that cluster analysis was more efficient than classic stability analysis. The most recent research involves GEI analysis, and that concept was part of our study, too, involving ANOVA, GGE (genotype main effect (G) plus genotype by environment interaction (GE)) AMMI Biplot analysis and correlations, using additional data to support primary field research in order to improve the efficiency of estimations [21]. The predictive accuracy of such research trials is described in previous work based on AMMI analyses [22]. It is essential to depict that all analysis methods must be properly selected for each kind of data, including raw data and stability indices [23].

The interaction of genotypes with the environment defined many different approaches to cope with stability problems [24] since a wide range of parameters affect cultivar behavior [25]. Al-Aysh et al. [26], assessing genotype by environment interaction, found a few stable pea lines, while Sayar [27] succeeded in revealing stable genotypes in common vetch (*Vicia sativa* L.).

AMMI analysis, which is the acronym of additive main effects and multiplicative interaction, is useful and commonly used in the estimation and evaluation of GEI. AMMI is a hybrid model that combines ANOVA and principal component analysis (PCA) and creates easily understandable figures regarding the GEI [10,28,29].

Gabriel [30] first reported the GGE (i.e., G + GE) biplot analysis, and it has since been applied in diverse topics such as economics, business, medicine, genetics, and ecology [31]. This method has been applied by many agricultural scientists for many crops [32,33,34,35,36]. Such an analysis can be conducted on data for traits and genotypes and depict their relationship, not only genotypes and environments [37].

Macák et al. [37] investigated the performance of field peas and conventional and low-input tillage. These researchers concluded that pea grain yield could be bolstered by incorporating the chloromass of the previous crop along with the application of fertilizer (to preserve performance in high levels). Hanáčková and Candráková [38] reported high yields in pea cultivations under low- or no-tillage conditions; however, they found that conventional treatments showed a higher protein content. Performance under low-input conditions must be taken into consideration because of the cultivation practices followed by farmers that use peas as feed for their livestock.

Peas are an interesting cultivar for animal feeding; thus, the highly productive varieties combined with a high quality of forage are preferable. The exploitation of the genetic parameters of the traits for breeding purposes is desirable. This knowledge is fundamental for effective breeding programs. Therefore, in order to initiate any breeding program, the exploitation of suitable parameters, such as the genotypic coefficient of variation GCV, heritability in a broad sense (H^2^) is necessary.

In the present study, the main scope was to determine the forage yield stability of pea genotypes along with various correlated traits based on the innovative approach of estimating the stability index, with the specific aim of studying pea genotypes’ behavior under both conventional and low-input cultivation systems. Greveniotis et al. [23,39] used a stability index, based on Fasoulas [7] and Fasoula [8] remarks, as an estimation of the heritability of various traits, leading to clear discrimination between qualitative and quantitative traits. Our approach includes stability performance analysis and reveals the stability performance and the kind of heritability of traits.

## 2. Results

### 2.1. ANOVA and Descriptive Statistics on the Stability Index

Regarding the ANOVA table (Table 1), the main effects for all traits expressed significant differences. The G × E interaction showed significant differences for all traits. Multiple interactions involving genotypes, the environment, and the cultivation system were found to be very significant, especially for forage yield, and these data must be analyzed in combination with the genotype performance within each environment and cultivation system in order to define the best genotype for specific conditions. Days to flowering showed no multiple interactions. Main stem length showed no cultivation × environment interaction. Days to flowering showed no multiple interactions.

To better analyze the performance of the genotypes in different environments and estimate the stability of each genotype for all traits, we used AMMI and GGE analysis as the most appropriate tools. For all traits, the GE was much more significant than G effects, and additional AMMI analysis was needed.

Stability estimations based on the calculation of the stability index of each trait are presented in Table 2, Table 3 and Table 4. The tabulated stability index data across environments for the seven characteristics under study are listed in Table 2. Days to 50% flowering and main stem length showed generally high indices (over 1000) in many cases. Fresh forage yield showed low indices. The low-input farming system seems to improve stability indices in many cases for forage yield.

In Table 3, the behavior of genotypes in all farming systems is shown. CultivarsPisso, Livioletta, and Vermio showed stability performance for forage yield and a few other traits, such as the main stem length and days to flowering—especially for Livioletta, which was generally more stable regarding forage yield.

Table 4 combines data for genotypes across environments and farming systems. This table is useful to depict the most stable genotype for a certain area (environment) and the selected farming system. In the Florina area and for forage yield, cv. Pisso displayed stable performance—the Giannitsa area stability index was over 600. Vermio was the best in the Trikala area (over 500), but only for low-input systems, not conventional.

Days to 50% flowering showed some extreme values, and for certain areas, it was 38,704. Generally, it was the most stable trait, with values over 10,000 in many cases. The main stem length showed a very stable behavior, with values over 1000.

Days to 50% flowering showed some extreme values for certain areas; for example, it was found to be 38,704 for cv. Pisso in Kalambaka (cv. Livioleta was 27,429). Generally, it was the most stable trait, with values over 10,000 in many cases. The main stem length showed a very stable behavior, with values over 1000. For some cultivars or lines, there were a few extreme values over 10,000 (line Zt2, cv. Olympos and cv. Vermio)—but also line Zt2, cv. Vermio in Kalambaka, cv. Olympos in Trikala, and cv. Livioletta in Giannitsa and in Florina, respectively, depending on the environment or cultivation method. Evidently, interactions led specific genotypes to exhibit varying behaviors for stability according to the environment or cultivation method.

### 2.2. The AMMI Tool for Multi-Environment Evaluations

The AMMI model is a widely used statistical tool in the analysis of multi-environment experiments. The purpose of the tool is to understand the complex GEI. In the AMMI model, the data are represented by a two-way table of GEI means. In complete tables, least-squares estimation is equivalent to fitting an additive two-way ANOVA model for the main effects and applying a single value decomposition to the interaction residuals [40].

Using this statistical tool, AMMI software can generate the adaptation map and AMMI1 biplot, where one axis is the axis of the factor, and the other is the PC1 value. When the PC1 value and its distance from the X-axis are close, the factor analyzed is stable. Regarding the AMMI1 biplot, the desirable genotypes were those with a high value on the axis of trait performance (*x*-axis, right position) and close to the center of the PC1 axis (near zero).

GGE stands for the genotype’s main effect (G) plus the genotype by environment interaction (GE), which is the only source of variation that is relevant to genotype evaluation. Mathematically, GGE is the genotype by environment data matrix after the environmental means are subtracted. A GGE biplot is a biplot that displays the GGE of a genotype through two-way environmental data. The GGE biplot methodology originates from the graphical analysis of multi-environment genotype trials (MET) data but is equally applicable to all other types of two-way data.

Regarding the GGE biplot for environments, the most stable environment was that placed close to the dot of the ideal and average environment and in the concentric area of the ideal environment dot. In terms of the GGE biplot for genotypes, the desirable genotypes (stable and productive) were those placed near the ideal genotype and in the concentric area of the ideal genotype dot.

The AMMI1 and G × E biplot analysis created biplots depicting the performance of the genotypes in different environments. The biplots created serve as a simple tool that can easily characterize each genotype for performance and stability.

**Figure 1:**
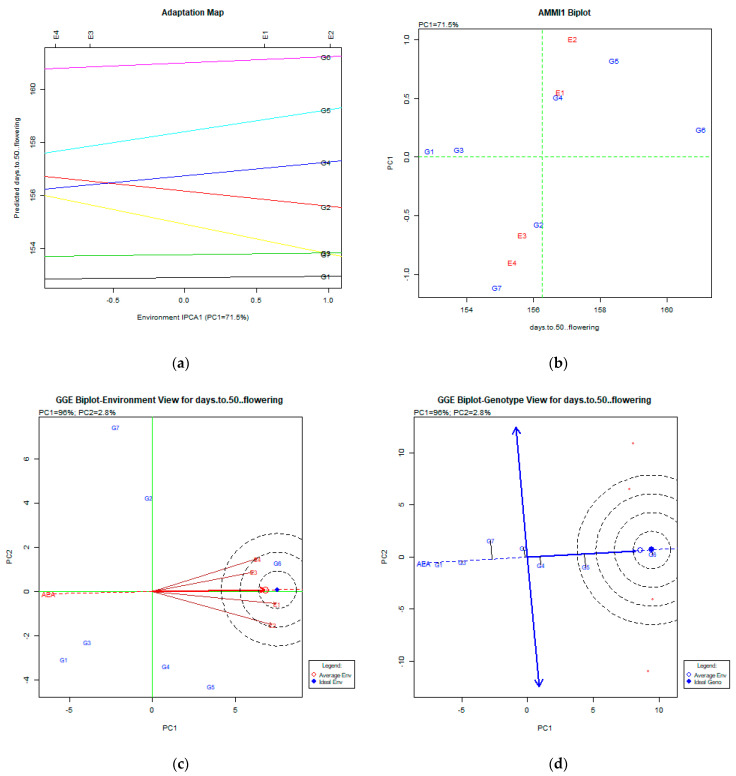
Stability analysis for days to 50% flowering based on (**a**) adaptation map, where the environmental stability of the genotypes is visualized by the *X*-axis (PC1) and the performance of the trait for the genotypes is tested by the *Y*-axis; (**b**) AMMI1 biplot, where the environmental stability of the genotypes is visualized by the *X*-axis (PC1) and the performance of the trait for the genotypes is tested by the *Y*-axis; (**c**) GGE biplot depicting the environmental stability over time for the desirable genotypes placed near to the ideal environment; (**d**) GGE biplot for genotypes depicting the genotypic stability in different environments. The desirable genotypes are those placed near the concentric region of the ideal genotype.

The stability analysis using both AMMI and GGE biplot for days to 50% flowering is depicted in Figure 1a–d.

**Figure 2:**
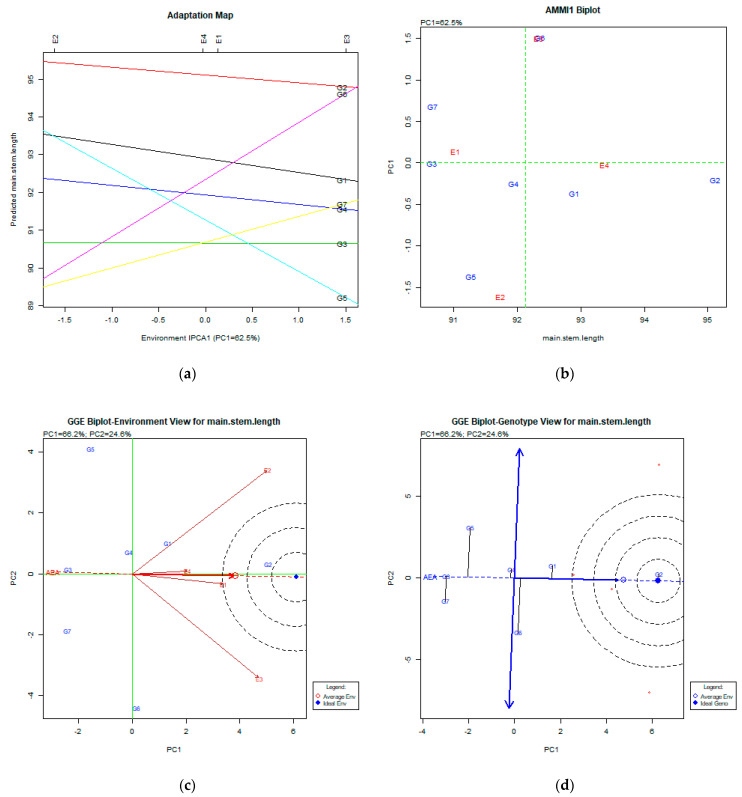
Stability analysis for the trait of main stem length (cm) based on (**a**) adaptation map, where the environmental stability of the genotypes is visualized by the *X*-axis (PC1) and the performance of the trait for the genotypes is tested by the *Y*-axis; (**b**) AMMI1 biplot, where the environmental stability of the genotypes is visualized by the *X*-axis (PC1) and the performance of the trait for the genotypes is tested by the *Y*-axis; (**c**) GGE biplot depicting the environmental stability over time for the desirable genotypes placed near the ideal environment; (**d**) GGE biplot for genotypes depicting the genotypic stability in different environments. The desirable genotypes are those placed near the concentric region of the ideal genotype.

The stability analysis using both AMMI and GGE biplot for main stem length is depicted in Figure 2a–d.

**Figure 3:**
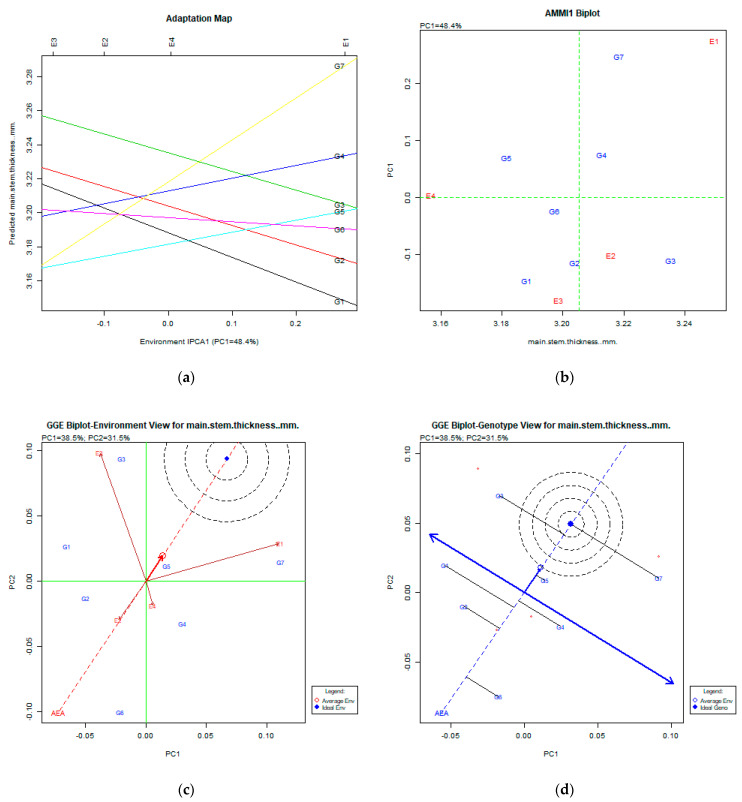
Stability analysis for the trait of main stem thickness (mm) based on: (**a**) adaptation map, where the environmental stability of the genotypes is visualized by the *X*-axis (PC1) and the performance of the trait for the genotypes is tested by the *Y*-axis; (**b**) AMMI1 biplot, where the environmental stability of the genotypes is visualized by the *X*-axis (PC1) and the performance of the trait for the genotypes is tested by the *Y*-axis; (**c**) GGE biplot depicting the environmental stability over time for the desirable genotypes placed near the ideal environment; (**d**) GGE biplot for genotypes depicting the genotypic stability in different environments. The desirable genotypes are those placed near the concentric region of the ideal genotype.

Data from the main stem thickness used in AMMI and GGE biplot analysis (Figure 3a–d) show that this trait was very environmentally dependent.

**Figure 4:**
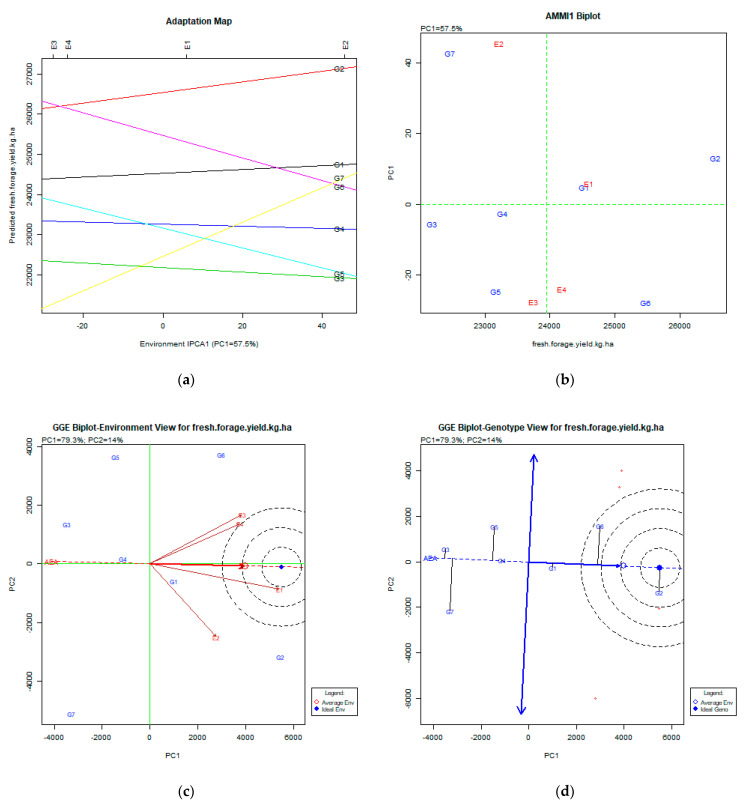
Stability analysis for the trait of fresh forage yield (kg ha^−1^) based on: (**a**) adaptation map, where the environmental stability of the genotypes is visualized by the *X*-axis (PC1) and the performance of the trait for the genotypes is tested by the *Y*-axis; (**b**) AMMI1 biplot, where the environmental stability of the genotypes is visualized by the *X*-axis (PC1) and the performance of the trait for the genotypes is tested by the *Y*-axis; (**c**) GGE biplot depicting the environmental stability over time for the desirable genotypes placed near to the ideal environment; (**d**) GGE biplot for genotypes depicting the genotypic stability in different environments. The desirable genotypes are those placed near the concentric region of the ideal genotype.

The stability analysis using both AMMI and GGE biplot for fresh forage yield is shown in Figure 4a–d.

**Figure 5:**
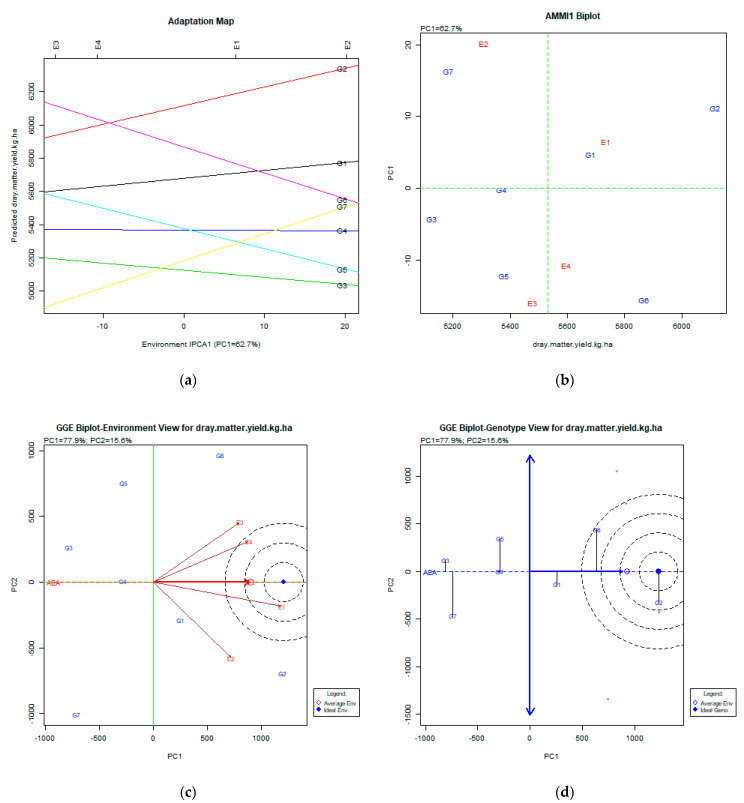
Stability analysis for the trait of dry matter yield (kg ha^−1^) based on: (**a**) adaptation map, where the environmental stability of the genotypes is visualized by the *X*-axis (PC1) and the performance of the trait for the genotypes is tested by the *Y*-axis; (**b**) AMMI1 biplot, where the environmental stability of the genotypes is visualized by the *X*-axis (PC1) and the performance of the trait for the genotypes is tested by the *Y*-axis; (**c**) GGE biplot depicting the environmental stability over time for the desirable genotypes placed near to ideal environment; (**d**) GGE biplot for genotypes depicting the genotypic stability in different environments. The desirable genotypes are those placed near the concentric region of the ideal genotype.

The stability analysis of dry matter yield using AMMI and GGE biplot is depicted in Figure 5a–d.

**Figure 6:**
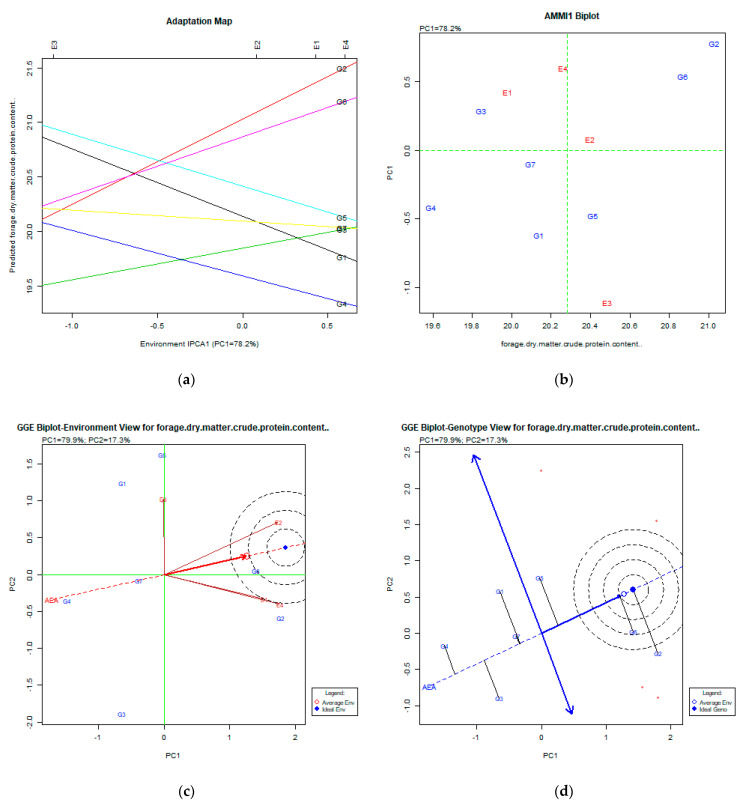
Stability analysis for the trait of forage dry matter crude protein content % based on: (**a**) adaptation map, where the environmental stability of the genotypes is visualized by the *X*-axis (PC1) and the performance of the trait for the genotypes is tested by the *Y*-axis; (**b**) AMMI1 biplot, where the environmental stability of the genotypes is visualized by the *X*-axis (PC1) and the performance of the trait for the genotypes is tested by the *Y*-axis; (**c**) GGE biplot depicting the environmental stability over time for the desirable genotypes placed near the ideal environment; (**d**) GGE biplot for genotypes depicting the genotypic stability in different environments. The desirable genotypes are those placed near the concentric region of the ideal genotype.

The stability analysis using AMMI and GGE biplot for forage dry matter crude protein content is presented in Figure 6a–d.

**Figure 7:**
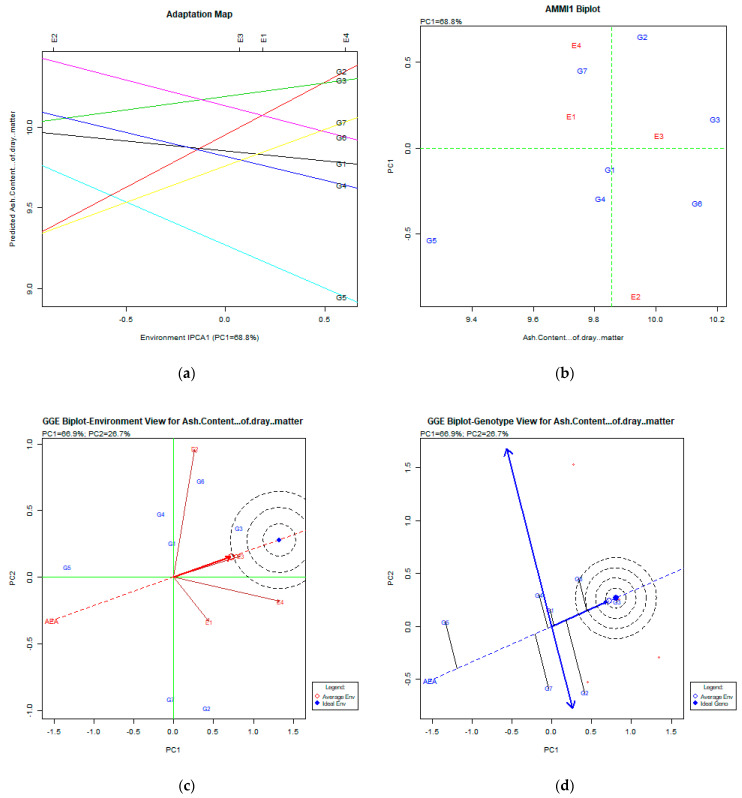
Stability analysis for the trait of ash content % of dry matter based on: (**a**) adaptation map, where the environmental stability of the genotypes is visualized by the *X*-axis (PC1) and the performance of the trait for the genotypes is tested by the *Y*-axis; (**b**) AMMI1 biplot, where the environmental stability of the genotypes is visualized by the *X*-axis (PC1) and the performance of the trait for the genotypes is tested by the *Y*-axis; (**c**) GGE biplot depicting the environmental stability over time for the desirable genotypes placed near the ideal environment; (**d**) GGE biplot for genotypes depicting the genotypic stability in different environments. The desirable genotypes are those placed near the concentric region of the ideal genotype.

The stability analysis for the ash content % of dry matter trait, using the AMMI and GGE biplot, is presented in Figure 7a–d.

For AMMI analysis, as visualized by the adaptation map figure, the most desirable genotypes were those placed high on the axis of trait performance, showing a nearly parallel line to the PC1 axis, which was an indication of stability in different environments.

For the AMMI1 biplot, the desirable genotypes were those placed high on the axis of trait performance (*x*-axis, right position) and close to the center of the PC1 axis (near the zero point).

Regarding the GGE biplot for environments, the most stable environment was that placed close to the dot of the ideal and average environment and in the concentric area of the ideal environment dot.

Concerning the GGE biplot for genotypes, the desirable genotypes (stable and productive) were those placed close to the ideal genotype and in the concentric area of the ideal genotype dot.

### 2.3. Exploratory Data Analysis of Peas

In order to estimate the phenotypic distances among genotypes, the clustering method of Ward was performed and formed clusters based on the traits tested. The clusters were formed based on the fresh forage yield and dry matter yield and the relations among them.

### 2.4. Genotypic and Phenotypic Coefficients of Variation and Heritability

In Table 5, estimations of genetic parameters for the traits are tabulated. The genetic parameters, along with the heritability in a broad sense, were estimated for all traits except the trait of main stem thickness. The parameters show that there is enough phenotypic variability for all traits. Furthermore, a large portion of phenotypic variability was genotypic, and this is desirable for geneticists in order to select superior genotypes for all traits. The heritability for all traits ranged from 99.4% to 83.8%. These estimates of heritability combined with the high percentage of genetic variability to the phenotype and the high diversity for all traits indicates that the selection of new varieties would be effective.

### 2.5. Correlations between All Characteristics

In Table 6, correlations between all traits are tabulated. Many correlations were statistically significant, especially between forage yield and traits such as the main stem length (r = 0.203), dry matter yield (r = 0.974), and forage dry matter crude protein content (r = 0.100).

## 3. Discussion

Farmers and breeders need both high and stable performance regarding forage yield. In our work, the two cultivation systems (conventional and low-input) displayed differences in genotype-yielding performance, but overall estimations on various pea characteristics seemed to be unaffected. In combination with GGE biplot analysis, the two farming systems revealed the most stable genotypes across all environments, as well as those more stable in specific environments and farming systems. Additionally, some genotypes exhibited stability in low-input conditions. Generally, very significant GGE interactions were recorded. Sayar and Han [41], based on ANOVA findings, state that G × E interaction is the most important concept to deal with. In our work, G × E interaction was revealed due to multiple interactions recorded for many traits. Sayar’s work [27] was based on AMMI analysis in order to define cultivar interactions with the environment. We described the interactions of each trait of pea cultivars and lines across different environments as follows:

### 3.1. Days to 50% Flowering

Regarding days to 50% flowering, AMMI analysis produced the figures adaptation map (Figure 1a) and AMMI1 biplot (Figure 1b). Both figures explained a portion of the total variability (71.5%), which is high enough for the genotype × environment (Gx) variation. Both the adaptation map and AMMI1 figures show that the most stable genotypes for environments E1 (Gianitsa) and E2 (Florina) were G6 (Zt1) and G5 (Dodoni), the late genotypes, whereas, for E3 (Trikala) and E4 (Kalambaka), the most stable genotypes were the early ones, G1 (Olympos), G2 (Pisso) and G7 (Zt1). The GGE analysis explained a total variability of 98.8% (PC1:96%, PC2: 2.8%), which was very high. The GGE biplot of the environment (Figure 1c) shows that all environments were quite similar and in the concentric circles of the ideal environment. The GGE biplot for the genotype view (Figure 1d) shows that all genotypes were very stable in all environments; the early genotypes were G1 (Olympos) and G2 (Pisso), and the late genotypes were G6 (Zt1) and G5 (Dodoni). The ideal for cultivation genotypes depends on what is desirable among early and late ones.

### 3.2. Main Stem Length (cm)

Regarding the main stem length, AMMI analysis produced the figures adaptation map (Figure 2a) and AMMI1 biplot (Figure 2b). Both figures explained a portion of the total variability (62.5%), which is high enough for conclusions. Both the adaptation map and AMMI1 figures show that the most stable genotypes were G2 (Pisso) and G1 (Olympos), where G2 (Pisso) had the highest performance for the main stem length trait. The GGE analysis explained a total variability of 90% (PC1:66.2%, PC2: 24.6%), which is very high. The GGE biplot for the environment view (Figure 2c) shows that all environments were very diverse, where the E1 (Giannitsa) environment was very close to the average environment. The GGE biplot for the genotype view (Figure 2d) shows that the most stable genotype and identical to the ideal genotype was G2 (Pisso), followed by the G1 (Olympos) genotype, which was very stable but with lower performance for this trait.

### 3.3. Main Stem Thickness (mm)

The AMMI analysis via the adaptation map (Figure 3a) and AMMI1 biplot expressed the PC1: 48.4% of the total variability. In both figures, there no clear pattern was found for stability, but the genotypes G3 (Livioletta), G4 (Vermio), and G2 (Pisso) were relatively stable. The GGE biplot analysis explained 70.0% (PC1:38.5%, PC2:31.5%) of the total variability. The GGE biplot for the environment view (Figure 3c) shows that all environments were very diverse, and no environment was placed near the average environment. The GGE biplot for the environment view (Figure 3d) shows that relative stable genotypes were G3 (Livioletta), G5 (Dodoni), and G7 (Zt2), but all were placed out of the concentric circles of the ideal genotype.

### 3.4. Fresh Forage Yield (kg ha^−1^)

The AMMI analysis explained a portion (57.1%) of PC1’s total variability. Both the adaptation map (Figure 4a) and AMMI1 biplot (Figure 4b) show that the relatively stable genotypes were G2 (Pisso), G6 (Zt1), and G1 (Olympos), while G2 (Pisso) had the highest fresh forage yield. The GGE biplot analysis expressed 93.3% (PC1:79.3%, PC2:14.0%) of the total variability. The GGE biplot for the environment view shows that E1 (Giannitsa) and E4 (Kalambaka) were close to the average environment, and all environments were very diverse. The GGE biplot for the genotype view shows that the most desirable genotype was G2 (Pisso), followed by G6 (Zt1), which was less stable, and G1 (Olympos), which was less productive than the other two but very stable. AMMI analysis assisted Sayar [27] in recommending the best cultivars for fresh forage yield in certain cultivation areas.

### 3.5. Dry Matter Yield (kg ha^−1^)

The AMMI analysis as presented from the adaptation map and AMMI1 biplot figures explained the PC1: 62.7% of the total variability. Both the adaptation map (Figure 5a) and AMMI1 biplot (Figure 5b) show that the most stable genotypes were G2 (Pisso), G6 (Zt1), and G1 (Olympos). The most productive genotype was G2 (Pisso), followed by G6 (Zt1) and G1 (Olympos). The GGE biplot analysis explained 95.5% (PC1:77.9%, PC2:15.6%) of the total variability. The GGE biplot for environment view shows that E1 (Giannitsa) and E4 (Kalambaka) were close to the average environment, and all environments were very diverse. The GGE biplot for the genotype view shows that the most desirable genotype was G2 (Pisso), followed by G6 (Zt1) which was less stable, and G1 (Olympos), which was less productive than the other two but very stable. Acikgoz et al. [14] investigated the dry matter, yield relations, and G × E interactions and concluded after a comparison of cluster and stability analyses that the stability analysis failed to recommend cultivars for different regions.

### 3.6. Forage Dry Matter Crude Protein Content %

The AMMI analysis explained the PC1: 78.2% of the total variability, which is quite high. Both the adaptation map (Figure 6a) and AMMI1 biplot (Figure 6b) figures show that the most productive genotypes were G2 (Pisso), G6 (Zt1), and G5 (Dodoni), which showed relatively low stability. The GGE biplot analysis explained 97.2% (PC1:79.9%, PC2:17.3%) of the total variability. The GGE biplot for the environment view shows that E2 (Florina), E1 (Giannitsa), and E4 (Kalambaka) were close to the average environment. The GGE biplot for the genotype view shows that the most desirable genotype was G2 (Pisso), G6 (Zt1), and G5 (Dodoni), which showed relatively low stability. Only the G6 (Zt1) genotype was placed in the concentric region of the ideal genotype, which indicates relatively acceptable stability and performance for this trait.

### 3.7. Ash Content % of Dry Matter

The AMMI analysis explained the PC1: 68.8% of the total variability, which is quite high. Both the adaptation map (Figure 7a) and AMMI1 biplot (Figure 7b) figures show that the most productive genotypes were G3 (Livioletta), followed by G6 (Zt1) and G2 (Pisso). The most stable one was the G3 (Livioletta) genotype. The GGE biplot analysis explained 93.6% (PC1:66.9%, PC2:26.7%) of the total variability. The GGE biplot for the environment view shows that E4 (Kalambaka) and E3 (Trikala) were close to the average environment. The GGE biplot for the genotype view shows that the most desirable genotypes were G3 (Livioletta) and G6 (Zt1), which showed relatively low stability. The G3 (Livioletta) genotype was placed nearly identical to the ideal genotype, which means that it has acceptable stability and performance for this trait.

### 3.8. Genotypic and Phenotypic Coefficients of Variation and Heritability

The traits of days to 50% flowering, the main stem length, main stem thickness, dry matter crude protein, and ash are components of fresh and dry forage yield. The traits of fresh forage yield and dry forage yield seem to have high variability, as described by the min and max of Table 5. The heritability estimate was 83.8% and 84.6%, respectively. These values are high [42]. The genetic variability and the GCV of these two traits are the highest among all other traits. This combination of high heritability (H^2^) and high GCV is an indication that the variation among genotypes was largely due to the additive genetic part [43]. Abebe et al. [44] suggested that high heritability in these values, in a broad sense, indicate that the characters under study are less influenced by the environment in their expression. This means that the direct selection of the traits of fresh forage yield and dry forage yield could be effective. As far as the other traits, the heritability (H^2^) was high, and the GCV was high to moderate, so the selection of these traits could be effective as well. The findings of this genetic analysis for the traits tested suggest that the selection of productive genotypes in order to create new varieties that are stable in all environments for conventional and organic cultivation is possible. The estimates of genetic parameters of forage dry matter crude protein content characterized by high heritability and high genetic variability, and GCV indicates that selecting for better quality, as described by the protein content, is possible.

### 3.9. Correlations between Traits

In our study, many correlations between traits displayed statistically significant results. Statistically significant correlations are useful for indirect breeding and selection of traits that show low stability through more stable traits that promote adaptation [7]. Positive correlations were also reported for other traits in common vetch and peas by Greveniotis et al. [2,45,46]. Georgieva et al. [47] reported significant correlations for many traits in field peas. We found positive relationships between the fresh forage yield and dry matter yield, which were expected, but also the main stem length and crude protein content.

Correlation studies are very important in the genetic improvement of cultivars [48,49]. Singh et al. [50] reported significant correlations between seed yield per plant and harvest index, as well as the biological yield per plant, plant height, number of seeds per pod, number of primary branches per plant, number of pods per plant, and 100-seed weight. Days to maturity and 100-seed weight and number of pods per plant showed a weak negative correlation with the seed yield per plant. In our results, the most interesting correlation was between the fresh forage yield and the stable characteristic, ‘stem length’, for indirect breeding purposes [46]. Linearity was not satisfactory in many cases due to low correlation coefficients. Cacan et al. [1] reported interesting yield performances for forage pea lines. They also reported statistically significant correlations between many traits studied. Kosev and Mikić [51] also reported high and significant correlations between many traits in peas and, most of all, with significant linearity.

Sayar and Han [41] used GGE biplot analysis in two growing seasons. Their results showed that two lines and cultivar Kirazli were superior for fresh forage yield, dry matter yield, plant height, and days to 50% flowering. PC2 scores of these genotypes were found near zero, making them stable genotypes. Bocianowski et al. [16] reported that AMMI analysis managed to depict certain cultivars for certain environments regarding seed yield. This was an encouraging result for practical farming.

Sayar and Han [41], as well as Yihunie and Gesesse [52], reported that the GGE biplot could be used as a tool for the discrimination of pea genotypes according to their productivity and stability and the selection of the most suitable genotype for cultivation.

Uzun et al. [53] assessed the dry matter performance for peas used for their forage yield. He reported that semi-leafless lines had significantly better standing ability than leafed peas. The leaf type had no effect on lodging scores at the seed-harvesting stage. Yihunie and Gesesse [52] used a GGE-biplot of field peas genotypes and defined the ideal genotype. Among the twelve environments used, three environments were the best for discrimination, while one genotype was found to be the most stable, the highest yielding, and it was recommended for wider cultivation in Northwestern Ethiopia and similar areas. Georgieva et al. [25] also reported the specific adaptation of certain genotypes in field peas. In our study, Vermio proved to be a stable genotype for forage yield performance in low-input farming in the Trikala and Kalambaka area, while Pisso was the best in Florina and Giannitsa areas and low-input farming systems. The two pea lines exhibited stable performance in Giannitsa and Florina areas, especially in low-input conditions. Livioletta was also a stable genotype.

### 3.10. Exploratory Data Analysis of Peas

**Figure 8:**
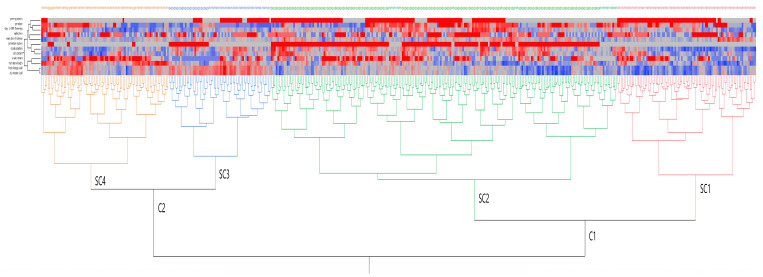
Dendrogram of two-way clustering based on the variables measured in peas using Ward’s method on the standardized data to define distances between clusters. Blue areas in the map dendrogram indicate low values, whereas the red areas indicate high values.

To provide a certain classification for the studied pea genotypes and cultivation systems, a heat map (Figure 8) was carried out. Cluster analysis was previously used for classification purposes for various genetic materials (e.g., maize, sweet cherry), sometimes in combination with principal components analysis [54,55,56]. The available data were divided into groups of increasing dissimilarity. Based on these results, the peas were divided into two distinct clusters (C1 and C2), each one having two subclusters (SC1, SC2, SC3, and SC4, respectively). Grouping for each subcluster revealed differences among pea cultivations. More specifically, SC1 consisted of low-input cultivated genotypes, which were characterized by a lower forage yield and dry matter yield, as well as lower ash content. SC2 included various other subgroups, mainly containing conventionally cultivated genotypes, which exhibited mostly low forage and dry matter yields. SC3 contained genotypes with higher yields, with two distinct subgroups, one cultivated conventionally and the other cultivated with low input. Lastly, SC4 included genotypes cultivated conventionally during the first growing season of experimentation and exhibiting higher main stems. There were no identified specific clusters based on locality.

## 4. Materials and Methods

### 4.1. Crop Establishment and Experimental Procedures

Four different locations (Table 7) were employed for the field experiments, two of them in Northern Greece and another two in Central Greece, divergent regarding soil type, altitude, and environmental conditions.

Five cultivars (common in Greek cultivations) of peas, namely, cv. Olympos, cv. Pisso, cv. Livioletta, cv. Vermio, and cv. Dodoni, and two lines (Zt1, Zt2) were used.

Two types of cultivation approaches were selected: low-input and conventional farming systems. The plots cultivated under the conventional farming system were fertilized before sowing so that 40 kg ha^−1^ Nitrogen and 80 kg ha^−1^ P_2_O_5_ were added into the soil. For low-input cultivation, no fertilizers or other agrochemicals were applied during the experiment, while prior to the establishment of the experiment in 2008, the fields had been in a two-year rotation consisting of bread wheat/legumes without nutritional supplementation or other agrochemical inputs. Weeds were fully controlled by hand.

All genotypes were sown in early November 2008 and 2009 according to a strip-plot design, with the seven genotypes randomized within each plot and a plot size of 8.75 m^2^. Replications were four for each plot. Each plot consisted of seven (7) rows 5 m long, spaced at 25 cm, and the number of plants per plot was around 1000 according to the sowing rate. The number of seeds was 120 per m^2^, and the depth of sowing was 4 cm.

### 4.2. Climatic Conditions

Experimentation lasted two growing seasons (2008–2009 and 2009–2010), and the mean monthly air temperatures (maximum, minimum, mean) and rainfall data during the study period are provided in Table 8 for each experimental area based on daily records.

### 4.3. Measurements

For each plot, the number of days from the sowing date to 50% of the flowering time was recorded. Ten random plants of each plot were selected at the flowering time and measured from the ground level to the top point with a ruler (1 mm sensitivity) after extending the plants upward. The arithmetic mean of the measurements (in cm) was accepted as the ‘main stem length’ for each plot. The main stem thickness (mm) was calculated by measuring the stem diameter at the top, middle, and bottom of each stem selected. These traits also served as correlation variables.

The chloromass (fresh forage) obtained from each plot right after harvesting in full flowering time was weighed, and the value was converted to a hectare basis in order to calculate the ‘fresh forage yield (kg ha^−1^)’. After, fresh forage samples (0.5 kg), harvested from each experimental plot, were placed in a drying oven at 70 °C for 48 h, left to cool, and weighed; the dry matter yield was determined for each plot, followed by the calculation on a hectare basis in order to obtain the ‘dry matter yield (kg ha^−1^)’.

In order to analyze the forage dry matter crude protein content (%) and ash content % of the dry matter, the forage dry matter was ground to pass through a 1 mm sieve and subsequently mixed for the analysis. Ash content was determined according to AOAC Official Method 942.05 [57], while total nitrogen was determined using AOAC Official Method 988.05 [57], followed by total protein content estimation.

### 4.4. Data Analysis

The experimental design was a combined analysis of seven genotypes in four replications over four locations for two cultivation systems and two years of experimentation. The formal ANOVA should include the interaction of years × locations or years × genotypes, etc., which were not the aims of our study and made no practical sense. To overcome such a problem, we created a simpler ANOVA with one degree of interaction less, and it did not affect the precision of the analysis for the genotypes in different environments, so we conducted an ANOVA as follows. In order for the ANOVA to be more informative, the combination of each year and location was assigned as an environment in the general meaning since locations and years contribute to the effect of the environment on the genotypes. In this way, we have fewer interactions in the ANOVA table and do not affect the variance of genotypes and the G × E (genotype × environment) interaction, which is crucial for proceeding in the stability analysis. Stability estimations were based on the stability index ${\left(\bar{x}/s\right)}^{2}$, where $\bar{x}$ and s are the entry mean trait and the standard deviation, respectively [8,58]. Trait correlations were examined using the Pearson coefficient according to Steel et al. [59], and the significance of all the statistics was checked at *p* < 0.05 using SPSS ver. 25. Stability analysis was performed using the free version of PB Tools v1.4 (International Rice Research Institute, Laguna, Philippines) over locations and years for each characteristic and the statistical tools were the AMMI1 and (GGE) biplot analysis.

The mean squared values of genotypes, genotype × environment, error, and replicates were used to estimate the variance components following the methods suggested by McIntosh [60], which were used for the estimation of genetic parameters for the tested traits as follows:

Heritability in a broad sense (H^2^) was calculated according to Johnson et al. [42] and Hanson et al. [61]:

$$H_{}^{2}=\frac{\sigma_{g}^{2}}{\sigma_{g}^{2}+\frac{\sigma_{gxe}^{2}{}_{}}{e}+\frac{\sigma_{re}^{2}}{rxe}}$$

The genotypic coefficient of variation (GCV) and phenotypic coefficient of variation (PCV) were calculated for all tested traits according to Singh and Chaudhary [62]:

$$\mathrm{GCV}(\%)=\frac{\sqrt{\sigma_{g}^{2}}}{\bar{x}}\times 100,$$

$$\mathrm{PCV}(\%)=\frac{\sqrt{\sigma_{p}^{2}}}{\bar{x}}\times 100$$

where $\sigma_{g}^{2}$, $\sigma_{p}^{2}$, $\sigma_{gxe}^{2}$, $\sigma_{re}^{2}$, and $\bar{x}$ are the genotypic variance, phenotypic variance, genotype × environment variance, residual variance (error), and overall mean for each tested trait, respectively.

The mathematical processing of the data was performed by hierarchical cluster analysis (HCA) using Ward’s method. HCA analysis was performed using JMP 14 (SAS Institute Inc., Cary, NC, USA). The results from the cluster analysis are presented in a dendrogram.

## 5. Conclusions

Correlations among various characteristics showed significant positive relationships between the forage yield along with the dry matter yield and forage dry matter crude protein content. Indirect forage yield stability improvement may be performed by improving the main stem length, which generally showed high stability indices.

Comparisons between conventional and low-input farming systems generally revealed genotypes that displayed highly stable performance, even in low-input farming systems. Stability index data could also serve to estimate the kind of heritability of various traits, either quantitative or qualitative.

AMMI analysis, and consequently, a GGE biplot, along with ANOVA data, showed that there is a strong interaction between genotypes and environments, as well as the farming system (conventional or low-input). Therefore, the necessity arises to propose certain genotypes of field peas for specific areas and farming systems so as to obtain the most stable performance. The Vermio cultivar proved to be a stable genotype for forage yield performance in low-input farming in Trikala and Kalambaka areas, while Pisso was the best in Florina and Giannitsa areas and low-input farming. The two pea lines displayed stable performance in Giannitsa and Florina areas, especially in low-input conditions. The stable behavior of some genotypes in low-input farming systems could be valuable for farmers that raise livestock in mountainous areas.

The genetic parameters showed that all traits were of high heritability and moderate to high GCV, and the direct selection for fresh forage yield and dry matter yield was expected to be effective.

Limitations of this study are related to the differences in environmental data through time (across years). Low rainfall may significantly affect the genotype behavior across different environments.

## Figures and Tables

**Table 1:** Mean squares (m.s.) from analysis of variance over environments and cultivation methods for tested traits: days to 50% flowering, main stem length (cm), main stem thickness (mm), fresh forage yield (kg ha^−1^), dry matter yield (kg ha^−1^), forage dry matter crude protein content (%), and ash content % of dry matter.

Source of Variation	Days to 50% Flowering	Main Stem Length (cm)	Main Stem Thickness (mm)	Fresh Forage Yield (kg ha^−1^)	Dry Matter Yield (kg ha^−1^)	Forage Dry Matter Crude Protein Content (%)	Ash Content % of Dry Matter
	m.s.	m.s.	m.s.	m.s.	m.s.	m.s.	m.s.
Environments (E)	163.29 **	68.52 **	0.09 **	28,290,714 **	1,938,835 **	3.95 **	3.71 **
REPS/Environments	1.16 ns	17.02 ns	0.01 ns	6,632,777 **	316,336 **	8.41 **	10.31 **
Genotypes (G)	499.53 **	155.90 **	0.02 *	168,425,535 **	8,653,394 **	17.54 **	5.92 **
Genotypes × Cultivation	2.01 *	48.89 *	0.04 **	102,070,746 **	5,364,623 **	1.18 **	0.44 **
Genotypes × Environments (G × E)	5.37 **	32.28 *	0.03 **	17,196,116 **	828,043 **	2.77 **	1.44 **
Cultivations	334.99 **	207.37 **	0.04 *	36,668,636 **	1,557,247 **	98.63 **	42.84 **
Cultivation × Environments	2.31 *	32.49 ns	0.08 **	31,475,811 **	1,614,424 **	0.36 **	20.09 **
Cultivation × Genotypes × Environments	1.03 ns	42.24 **	0.04 **	26,630,013 **	1,270,305 **	0.29 **	0.18 **
Error	0.95	2.128	0.01	1,609,523	69,682	0.020	0.03

Probability values: * *p* ≤ 0.05; ** *p* ≤ 0.01; ns = not significant.

**Table 2:** Trait stability index across environments for two farming systems: days to 50% flowering, main stem length (cm), main stem thickness (mm), fresh forage yield (kg ha^−1^), dry matter yield (kg ha^−1^), forage dry matter crude protein content (%), and ash content % of dry matter.

	Environments	Days to 50% Flowering	Main Stem Length (cm)	Main Stem Thickness (mm)	Fresh Forage Yield (kg ha^−1^)	Dry Matter Yield (kg ha^−1^)	Forage Dry Matter Crude Protein Content %	Ash Content % of Dry Matter
Conventional	Giannitsa	1735	544	301	41	44	379	117
	Florina	2127	675	533	59	57	284	108
	Trikala	3391	927	1012	38	44	402	115
	Kalambaka	3173	2726	3009	57	57	357	83
Low-input	Giannitsa	2091	1816	484	60	61	449	119
	Florina	2142	1344	664	94	126	438	123
	Trikala	2987	2575	422	68	79	446	130
	Kalambaka	2461	1531	649	120	148	435	109
Conventional and Low-input	Giannitsa	1824	720	367	49	51	343	104
	Florina	2074	905	593	73	79	307	107
	Trikala	2881	1348	593	46	53	340	113
	Kalambaka	2458	1860	1065	73	80	315	85

**Table 3:** Trait stability index across genotypes for the two farming systems: days to 50% flowering, main stem length (cm), main stem thickness (mm), fresh forage yield (kg ha^−1^), dry matter yield (kg ha^−1^), forage dry matter crude protein content (%), and ash content % of dry matter.

	Genotypes	Days to 50% Flowering	Main Stem Length (cm)	Main Stem Thickness (mm)	Fresh Forage Yield (kg ha^−1^)	Dry Matter Yield (kg ha^−1^)	Forage Dry Matter Crude Protein Content %	Ash Content % of Dry Matter
Conventional	Olympos	4766	888	1124	97	121	354	133
	Pisso	7190	2155	1735	140	148	475	115
	Livioletta	7544	1661	388	135	135	459	133
	Vermio	5127	1650	738	88	99	533	111
	Dodoni	4208	1539	881	58	64	453	81
	Zt1	3881	579	476	68	66	540	123
	Zt2	5786	847	364	84	86	526	118
Low-input	Olympos	5693	1885	690	87	108	472	146
	Pisso	7613	1751	1016	51	52	486	129
	Livioletta	8642	1286	408	139	204	471	118
	Vermio	7799	1542	362	125	193	438	116
	Dodoni	5006	2130	476	59	55	623	122
	Zt1	8035	993	343	151	202	541	153
	Zt2	10,911	3094	638	64	71	544	107
Conventional and Low-input	Olympos	4487	1194	846	93	115	325	126
	Pisso	6254	1731	1292	46	48	460	119
	Livioletta	6589	1413	395	114	130	347	118
	Vermio	5305	1462	473	83	98	390	104
	Dodoni	4090	1648	586	59	60	407	84
	Zt1	4080	630	386	85	86	461	120
	Zt2	6836	1347	468	71	75	337	95

**Table 4:** Combined trait stability index across genotypes and environments for the two farming systems: days to 50% flowering, main stem length (cm), main stem thickness (mm), fresh forage yield (kg ha^−1^), dry matter yield (kg ha^−1^), forage dry matter crude protein content (%), ash content % of dry matter.

	Genotypes	Days to 50% Flowering	Main Stem Length (cm)	Main Stem Thickness (mm)	Fresh Forage Yield (kg ha^−1^)	Dry Matter Yield (kg ha^−1^)	Forage Dry Matter Crude Protein Content %	Ash Content % of Dry Matter
	Giannitsa							
Conventional	Olympos	3420	5714	591	294	507	436	134
	Pisso	4193	9510	1778	569	437	600	130
	Livioletta	6005	800	239	147	247	574	186
	Vermio	5724	933	834	71	73	663	75
	Dodoni	3471	2586	412	30	32	510	71
	Zt1	2905	8231	293	44	43	864	147
	Zt2	4254	2239	298	98	97	786	143
Low-input	Olympos	4118	2902	760	130	140	402	146
	Pisso	6867	6588	411	633	720	893	143
	Livioletta	7437	13,802	327	439	470	598	100
	Vermio	6470	9720	684	90	132	461	81
	Dodoni	5693	26,290	1184	24	23	921	124
	Zt1	5386	8439	430	399	528	704	133
	Zt2	7046	6798	1474	82	59	598	128
Conventional and Low-input	Olympos	3600	2443	684	190	233	315	133
	Pisso	4795	4764	708	538	509	619	124
	Livioletta	5997	1621	289	88	107	424	123
	Vermio	5870	467	406	64	74	475	79
	Dodoni	4228	2573	577	29	28	464	81
	Zt1	3668	268	186	56	55	692	130
	Zt2	4391	2417	355	81	65	491	101
	Florina							
Conventional	Olympos	4556	1110	1957	178	196	590	148
	Pisso	4709	1626	3689	791	513	746	96
	Livioletta	7338	11,783	350	146	139	603	104
	Vermio	8348	3337	358	269	442	511	186
	Dodoni	5397	3092	729	174	170	706	87
	Zt1	4315	4055	399	215	240	643	145
	Zt2	9480	4485	725	98	124	386	90
Low-input	Olympos	7142	1362	2903	31	39	734	162
	Pisso	5487	1700	2236	307	444	375	109
	Livioletta	8024	2574	941	149	511	470	140
	Vermio	11,714	3077	404	113	299	549	151
	Dodoni	7228	3662	801	55	78	967	127
	Zt1	4205	9218	326	291	395	812	166
	Zt2	9946	4165	499	284	329	823	116
Conventional and Low-input	Olympos	5354	849	2451	57	70	486	144
	Pisso	5101	1705	1225	95	93	531	104
	Livioletta	7078	4333	506	97	122	309	107
	Vermio	8853	2523	381	114	168	469	155
	Dodoni	5516	3340	746	78	104	596	102
	Zt1	4151	3239	384	248	311	632	153
	Zt2	8990	2881	592	149	192	397	101
	Trikala							
Conventional	Olympos	8945	3180	2478	32	39	485	183
	Pisso	11,898	2917	1701	79	75	327	178
	Livioletta	20,733	3844	496	268	178	387	169
	Vermio	11,755	4665	1098	114	172	549	107
	Dodoni	11,729	1154	2544	86	178	558	129
	Zt1	16,981	1810	1666	114	112	527	92
	Zt2	10,124	17,502	2158	75	102	460	150
Low-input	Olympos	5348	12,101	854	375	486	772	144
	Pisso	9580	908	2804	256	267	450	128
	Livioletta	12,243	7686	1106	64	109	236	155
	Vermio	13,050	12,343	228	569	796	936	97
	Dodoni	12,591	3145	201	479	391	796	121
	Zt1	24,270	3716	901	196	339	476	132
	Zt2	22,794	8975	351	109	82	396	139
Conventional and Low-input	Olympos	5520	4435	640	63	78	506	145
	Pisso	8503	1122	2079	18	20	380	159
	Livioletta	10,698	2202	680	110	143	255	169
	Vermio	8453	7203	393	81	102	417	101
	Dodoni	9319	1216	374	142	225	515	111
	Zt1	9155	2563	1007	155	176	465	103
	Zt2	11,099	12,133	647	72	76	243	103
	Kalambaka							
Conventional	Olympos	11,945	1617	1364	222	275	885	90
	Pisso	38,704	2185	8494	100	112	902	112
	Livioletta	27,429	2785	2088	190	241	463	176
	Vermio	8138	13,133	4768	103	110	531	121
	Dodoni	16,768	9926	3175	121	163	856	162
	Zt1	13,542	5841	3894	163	188	463	169
	Zt2	3435	12,901	3572	58	51	507	87
Low-input	Olympos	9304	1443	678	125	164	897	146
	Pisso	8098	4612	1367	297	545	781	156
	Livioletta	14,546	442	189	361	419	844	98
	Vermio	11,685	6158	777	317	470	518	160
	Dodoni	7719	2190	958	195	463	873	121
	Zt1	21,627	4766	1075	177	264	804	152
	Zt2	12,166	3613	1116	47	54	952	72
Conventional and Low-input	Olympos	6814	1591	966	153	203	516	110
	Pisso	8481	2977	2505	62	71	681	132
	Livioletta	10,069	815	372	227	231	496	128
	Vermio	6461	7037	1340	166	180	452	114
	Dodoni	7493	3521	1517	151	188	564	86
	Zt1	4138	3336	1756	80	104	431	117
	Zt2	5731	3069	1694	53	55	334	77

**Table 5:** Estimations of genetic parameters for tested traits: days to 50% flowering, main stem length (cm), main stem thickness (mm), fresh forage yield (kg ha^−1^), dry matter yield (kg ha^−1^), forage dry matter crude protein content (%), and ash content % of dry matter.

Traits	Min	Max	Mean	sd	$\sigma_{g}^{2}$	$\sigma_{p}^{2}$	GCV (%)	PCV (%)	H^2^ (%)
Days to 50% flowering	148.1	165.7	156.3	3.38	7.76	7.81	1.78	1.79	99.4
Main stem length (cm)	85.2	99.8	92.3	2.98	2.04	2.31	1.55	1.65	88.4
Main stem thickness (mm)	2.78	3.76	3.21	0.14	-	-	-	-	-
Fresh forage yield (kg ha^−1^)	17,257	34,932	23,947	3201	2,205,760	2,631,649	6.20	6.77	83.8
Dry matter yield (kg ha^−1^)	4080	7930	5531	714.2	114,321	135,209	6.11	6.48	84.6
Forage dry matter crude protein content (%)	17.8	23.3	20.3	1.14	0.251	0.274	2.47	2.58	91.4
Ash content % of dry matter	7.29	12.45	9.9	0.98	0.081	0.093	2.87	3.08	87.0

sd—standard deviation, $\sigma_{g}^{2}$—genotypic variance, $\sigma_{p}^{2}$—phenotypic variance, GCV—genotypic coefficient of variation, PCV—phenotypic coefficient of variation, and H^2^—broad sense heritability (%).

**Table 6:** Correlations between all traits measured: days to 50% flowering, main stem length (cm), main stem thickness (mm), fresh forage yield (kg ha^−1^), dry matter yield (kg ha^−1^), forage dry matter crude protein content (%), and ash content % of dry matter.

	Days to 50% Flowering	Main Stem Length (cm)	Main Stem Thickness (mm)	Fresh Forage Yield (kg ha^−1^)	Dry Matter Yield (kg ha^−1^)	Forage Dry Matter Crude Protein Content %
Main stem length (cm)	−0.061					
Main stem thickness (mm)	−0.004	−0.231 **				
Fresh forage yield (kg ha^−1^)	0.032	0.203 **	0.016			
Dry matter yield (kg ha^−1^)	0.028	0.210 **	0.009	0.974 **		
Forage dry matter crude protein content (%)	0.289 **	0.006	0.004	0.100 *	0.084	
Ash content % of dry matter	0.100 *	−0.050	0.048	−0.084	−0.091	0.676 **

* Correlations significant at the 0.05 level (2-tailed), ** Correlation is significant at the 0.01 level (2-tailed).

**Table 7:** Coordinates, altitude, soil type, and cultivation information for the environments of the experiment.

Environments	Longitude	Latitude	Elevation (m)	Soil Texture	Planting Date	Harvesting Date
Giannitsa	22°39′ E	40°77′ N	10	Clay (C)	Early November 2008 and 2009	Late May 2009 and 2010
Florina	21°22′ E	40°46′ N	705	Sandy loam (SL)	Early November 2008 and 2009	Late May 2009 and 2010
Trikala	21°64′ E	39°55′ N	120	Sandy clay loam (SCL)	Early November 2008 and 2009	Late May 2009 and 2010
Kalambaka	21°65′ E	39°64′ N	190	Silty clay (SiC)	Early November 2008 and 2009	Late May 2009 and 2010

**Table 8:** Climatic conditions for the examined environments during the cultivation period (November–May).

Year and Environments	Mean Monthly MaximumTemperature (°C)	Mean Monthly Minimum Temperature (°C)	Mean Temperature (°C)	Rainfall (mm)
Giannitsa 2008–2009	22.9	−0.1	10.5	51.5
Giannitsa 2009–2010	23.3	0.6	11.0	55.5
Florina 2008–2009	21.0	−5.7	7.1	44.1
Florina 2009–2010	21.3	−3.8	8.0	62.3
Trikala 2008–2009	23.4	1.3	11.0	55.8
Trikala 2009–2010	24.4	2.9	12.0	85.1
Kalambaka 2008–2009	21.1	0.4	10.8	68.4
Kalambaka 2009–2010	23.5	2.2	11.7	98.8

## Data Availability

The datasets used and/or analyzed during the current study are available from the corresponding author on reasonable request.
